# Pediatric-Onset Multiple Sclerosis Presenting With Persistent Vertigo, Ataxia, and Optic Nerve Atrophy in a 15-Year-Old Girl: A Case Report

**DOI:** 10.7759/cureus.103711

**Published:** 2026-02-16

**Authors:** Victor Hugo Spitz, Ruilin Wang, Anna DeBonaventura, Anusri Pakhare, Jessica N Smock, Kristie Rivers

**Affiliations:** 1 Medical School, Nova Southeastern University, Fort Lauderdale, USA; 2 Pediatrics, Broward Health Medical Center, Fort Lauderdale, USA

**Keywords:** central vertigo, dawson’s fingers, demyelinating disease, gait ataxia, nystagmus, optic nerve atrophy, optic neuritis, pediatric-onset multiple sclerosis, periventricular white matter lesions, persistent vertigo

## Abstract

Pediatric-onset multiple sclerosis (POMS) is an inflammatory demyelinating disorder of the central nervous system that can present with brainstem or cerebellar symptoms and may be initially misattributed to peripheral vestibular etiologies. We report a 15-year-old girl with five days of continuous vertigo described as a sensation that "everything around me is moving," accompanied by unsteady gait and transient unilateral sensory-motor symptoms. Brain magnetic resonance imaging (MRI) with and without contrast demonstrated extensive supratentorial and infratentorial T2-weighted fluid-attenuated inversion recovery (T2/FLAIR) hyperintense lesions in a periventricular distribution with corpus callosum involvement and lesions morphologically suggestive of Dawson’s fingers, with both enhancing and non-enhancing plaques. MRI of the orbits showed right-greater-than-left optic nerve atrophy with increased T2 signal without enhancement, consistent with sequelae of prior optic neuritis. Cervical and thoracic spine MRI revealed multiple short-segment T2-weighted hyperintense cord lesions, including an enhancing lesion at C5-C6 compatible with active demyelination. Cerebrospinal fluid (CSF) analysis demonstrated mild pleocytosis, mildly elevated protein, mildly decreased glucose, elevated immunoglobulin G (IgG) index and IgG synthesis rate, and CSF-restricted oligoclonal bands. The patient was treated with five days of high-dose intravenous methylprednisolone followed by an oral corticosteroid taper with significant clinical improvement. This case highlights the importance of considering POMS in adolescents with persistent vertigo and central ocular motor findings, and demonstrates the diagnostic value of combined brain/orbit/spine MRI and CSF immunologic studies for establishing dissemination in space and time and excluding important mimics.

## Introduction

Multiple sclerosis (MS) is a chronic immune-mediated demyelinating disease of the central nervous system. When symptom onset occurs before 18 years of age, the condition is referred to as pediatric-onset multiple sclerosis (POMS) [1,2]. Compared with adult-onset MS, POMS often presents with more inflammatory activity at onset and may manifest with brainstem or cerebellar features that can mimic peripheral vestibular disorders, migraine variants, or cerebrovascular events [3,4]. Early recognition is critical because timely treatment of acute relapses and initiation of long-term disease-modifying therapy can reduce relapse frequency and limit disability accumulation [3,4]. We describe an adolescent who presented with persistent vertigo, gait instability, and visual symptoms, whose neuroimaging demonstrated multifocal demyelinating lesions in the brain and spinal cord with concurrent enhancing and non-enhancing plaques, as well as optic nerve atrophy suggestive of a prior optic neuritis event.

## Case presentation

History of present illness

A 15-year-old girl with a history of attention-deficit/hyperactivity disorder (ADHD) presented with five days of continuous dizziness that she described as a spinning sensation with the perception that “everything around me is moving.” One day prior to symptom onset, she had a single episode of emesis. She also reported right upper arm soreness and transient left-sided weakness for approximately one week. These symptoms were accompanied by intermittent subjective “pushback” when lifting with the right arm and episodic involuntary twitching confined to the right upper extremity, occurring primarily after exposure to cold temperatures, without generalized involvement. She additionally endorsed transient sensory symptoms described as brief episodes of numbness and paresthesias involving the left upper extremity. She reported unsteady gait during this period.

Notably, she recalled an episode approximately one year earlier characterized by headache, vomiting, and significant balance difficulties for which she was evaluated at an outside hospital; a head computed tomography study was reportedly normal, and she did not receive neurology follow-up. Since that episode, she reported persistent baseline gait unsteadiness that never fully resolved prior to the current presentation.

Past medical history

The patient’s past medical history was notable for ADHD. She was not receiving chronic pharmacologic therapy at the time of presentation. There was no known history of prior demyelinating disease, autoimmune disease, or recent infection documented at presentation.

Examination

On arrival, she was afebrile and hemodynamically stable. Initial examination was notable for complaints of right upper arm pain and unsteady gait. During hospitalization, she was observed to have nystagmus and reported subjective visual disturbances described as intermittent blurred vision and difficulty focusing, without frank diplopia, visual field deficits, or acute vision loss. A general physical examination was otherwise unremarkable. At discharge, she was alert and oriented without focal neurologic deficits and demonstrated improved gait stability.

Laboratory studies

Initial laboratory studies including complete blood count, comprehensive metabolic panel, erythrocyte sedimentation rate, C-reactive protein, and serum glucose were within reference ranges. Additional testing included urine toxicology, vitamin B12 level, respiratory viral testing, and an extensive autoimmune/serologic panel. A lumbar puncture was performed as part of the demyelinating disease evaluation. Cerebrospinal fluid (CSF) analysis demonstrated a mild leukocytosis with lymphocytic predominance, mildly decreased glucose, and mildly elevated protein. CSF immunologic testing revealed elevated immunoglobulin G (IgG) index and IgG synthesis rate and CSF-restricted oligoclonal bands, supporting intrathecal IgG production. CSF culture was negative. Myelin oligodendrocyte glycoprotein (MOG) antibody testing in CSF was negative.

Table 1 summarizes selected laboratory findings relevant to the evaluation of inflammatory demyelinating disease, and Table 2 provides a brief clinical timeline.

**Table 1:** Cerebrospinal fluid and selected laboratory findings Cerebrospinal fluid (CSF) analysis demonstrated mild lymphocytic pleocytosis, mildly decreased glucose, and mildly elevated protein. Immunologic studies showed elevated CSF IgG concentration, IgG index, and IgG synthesis rate with CSF-restricted oligoclonal bands, supporting intrathecal IgG production consistent with an inflammatory demyelinating process. CSF myelin oligodendrocyte glycoprotein (MOG) antibody testing was negative. Serum vitamin B12 level was within the reference range, helping exclude a nutritional etiology for demyelination.

Test	Result	Comment/Reference
CSF white blood cells	8 cells/µL	Lymphocyte predominant (90% lymphocytes, 10% monocytes)
CSF red blood cells	2 cells/µL	
CSF glucose	57 mg/dL	Mildly decreased
CSF protein	63.7 mg/dL	Mildly elevated
CSF IgG	10.3 mg/dL	Elevated (0.8–7.7 mg/dL)
CSF IgG index	1.13	Elevated (<0.70)
CSF IgG synthesis rate	26.9 mg/24 h	Elevated
Oligoclonal bands (CSF)	Present	CSF-restricted; >5 bands reported
MOG antibody (CSF)	Negative	
Vitamin B12	334 pg/mL	Within reference range

**Table 2:** Clinical timeline. This table summarizes the patient’s clinical course, highlighting a prior self-limited neurologic episode approximately one year before presentation, followed by the current admission for persistent vertigo and gait instability. Key diagnostic milestones included brain, orbital, and spinal MRI demonstrating disseminated demyelinating lesions and cerebrospinal fluid (CSF) findings supportive of intrathecal IgG synthesis. The timeline also outlines acute treatment with high-dose intravenous corticosteroids and subsequent clinical improvement, culminating in discharge with a prolonged oral steroid taper and referral to a tertiary multiple sclerosis center for long-term management.

Time point	Key events
~1 year prior	Episode of headache, vomiting, and balance difficulty; reported normal head computed tomography (CT) at outside facility; persistent imbalance thereafter.
Day 0 (presentation)	Five days of continuous vertigo with unsteady gait and transient sensory-motor symptoms; initial blood work reportedly within reference ranges.
Hospital day 1	Brain magnetic resonance imaging (MRI) demonstrated extensive periventricular/callosal and infratentorial lesions with both enhancing and non-enhancing plaques; magnetic resonance venography negative for venous sinus thrombosis.
Hospital day 1–2	Ophthalmology evaluation for visual changes; orbital MRI showed right-greater-than-left optic nerve atrophy consistent with prior optic neuritis.
Hospital day 2	Lumbar puncture performed; CSF showed mild pleocytosis, elevated IgG index/synthesis rate, and oligoclonal bands. Cervical and thoracic spine MRI showed multiple short-segment cord lesions including an active enhancing cervical lesion.
Hospital days 2–6	Treated with high-dose intravenous (IV) methylprednisolone for five days with marked improvement in dizziness, nystagmus, and gait.
Discharge	Discharged on prolonged oral steroid taper with gastrointestinal prophylaxis; outpatient follow-up arranged with tertiary multiple sclerosis center for long-term management.

Imaging

Brain magnetic resonance imaging (MRI) with and without contrast demonstrated extensive supratentorial and infratentorial T2-weighted fluid-attenuated inversion recovery (T2/FLAIR) hyperintensities, most prominent in the periventricular white matter oriented perpendicular to the ventricles with corpus callosum involvement (Dawson’s finger morphology). Many lesions were hypointense on T1-weighted imaging (suggesting chronic axonal injury). Multiple enhancing lesions were present, including an incomplete ring-enhancing 1.0 cm lesion in the left centrum semiovale, a 0.4 cm nodular enhancing focus in the right centrum semiovale, an incomplete ring-enhancing 0.6 cm lesion in the right basal ganglia, and a ring-enhancing lesion in the right middle cerebellar peduncle measuring 0.7 cm. An enhancing lesion was also described in the upper cervical spinal cord at the C5-C6 level measuring 1.2 cm craniocaudally. No restricted diffusion was present to suggest acute infarction. Magnetic resonance venography showed no evidence of dural venous sinus thrombosis. MRI of the orbits demonstrated right-greater-than-left optic nerve atrophy with increased T2 signal and no definite optic nerve enhancement, interpreted as sequelae of prior optic neuritis. Cervical spine MRI revealed numerous additional non-enhancing short-segment T2 hyperintense cord lesions from C1 through C6, with an enhancing lesion at C5-C6 compatible with active demyelination. Thoracic spine MRI showed a short-segment T2 hyperintense cord lesion at T4-T5 without abnormal enhancement. Lumbar spine MRI was normal.

**Figure 1:**
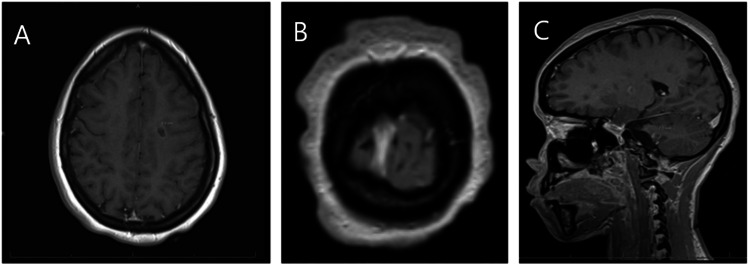
Brain MRI demonstrating multifocal enhancing supratentorial and infratentorial demyelinating lesions consistent with active multiple sclerosis. Brain MRI. (A) Axial T1-weighted post-contrast image demonstrating an enhancing demyelinating plaque. (B) Additional axial brain MRI slice highlighting a focal lesion. (C) Sagittal T1-weighted post-contrast image demonstrating an enhancing infratentorial plaque (measurement shown), consistent with active demyelination.

**Figure 2:**
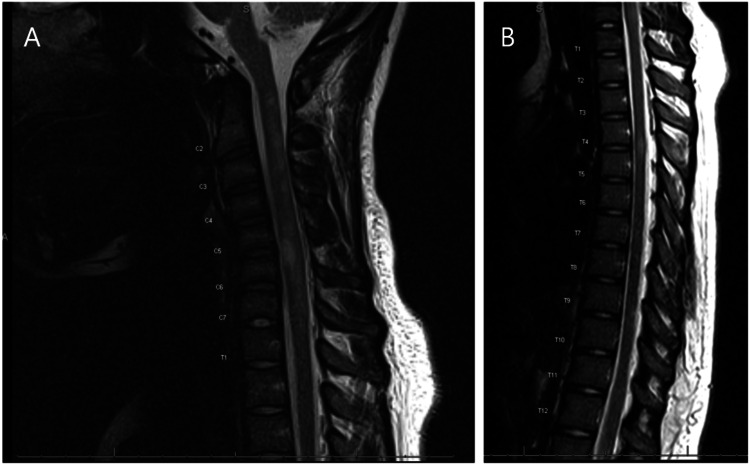
Spine MRI demonstrating multifocal short-segment cervical and thoracic spinal cord demyelinating lesions. Spine MRI. (A) Sagittal T2-weighted cervical spine image demonstrating multifocal short-segment intramedullary T2 hyperintense lesions with an active enhancing lesion reported at C5–C6. (B) Sagittal T2-weighted thoracic spine image demonstrating a short-segment T2 hyperintense cord lesion at T4–T5 without enhancement.

Figure 1 demonstrates representative brain MRI findings, and Figure 2 demonstrates spinal cord involvement.

Hospital course and follow-up

Given concern for an inflammatory demyelinating disorder, pediatric neurology recommended brain MRI with and without contrast. After imaging demonstrated disseminated demyelination, ophthalmology was consulted for visual symptoms and recommended orbital MRI. Additional evaluation included magnetic resonance venography and lumbar puncture, and spine MRI was obtained to further characterize demyelinating burden. The patient received five days of high-dose intravenous methylprednisolone. Physical therapy assisted with gait and balance rehabilitation. Palliative care was consulted for symptom management, and psychology was consulted to support coping with a new chronic disease diagnosis. By the third day of corticosteroid therapy, dizziness and sensory symptoms improved and nystagmus was no longer observed. She completed the planned steroid course with significant clinical improvement and was discharged home on a prolonged oral corticosteroid taper with gastrointestinal prophylaxis. Outpatient follow-up with a tertiary MS center was arranged for long-term management.

## Discussion

This case illustrates a classic radiographic and laboratory profile of POMS presenting with a vestibular-cerebellar syndrome. The patient’s continuous vertigo, nystagmus, and gait instability suggested central involvement, and MRI confirmed multifocal supratentorial and infratentorial lesions. The presence of periventricular plaques oriented perpendicular to the ventricles with corpus callosum involvement and Dawson’s finger morphology is highly characteristic of MS [1]. Concurrent enhancing and non-enhancing lesions provided evidence of dissemination in time, while lesions in multiple anatomic locations (periventricular, infratentorial, and spinal cord) supported dissemination in space [1,2]. The absence of diffusion restriction and the presence of characteristic demyelinating morphology helped distinguish this presentation from posterior circulation ischemia, an uncommon but important consideration in adolescents with central vertigo. Several important demyelinating disorders can mimic POMS in children and adolescents. Acute disseminated encephalomyelitis (ADEM) is typically monophasic and more often associated with encephalopathy; callosal Dawson-finger morphology and a mix of enhancing and non-enhancing lesions argue against ADEM [2,5-7]. Myelin oligodendrocyte glycoprotein antibody-associated disease (MOGAD) can present with optic neuritis and myelitis; however, MOG antibody testing was negative in this patient, and the spinal cord lesions were short-segment rather than longitudinally extensive [5]. Neuromyelitis optica spectrum disorder (NMOSD) is another critical consideration in pediatric demyelination, classically associated with severe optic neuritis and longitudinally extensive transverse myelitis; this patient’s imaging pattern and serologic workup did not support NMOSD [6,7]. A notable feature in this case is MRI evidence of optic nerve atrophy without enhancement, interpreted as sequelae of prior optic neuritis. The patient’s history of a prior episode of headache, vomiting, and balance difficulty approximately one year earlier may represent an unrecognized demyelinating event, consistent with the relapsing-remitting course typical of MS. Optic neuritis and brainstem/cerebellar involvement are common initial manifestations of POMS [3,4]. High-dose intravenous corticosteroids remain first-line therapy for acute MS relapses in pediatric patients, and this patient demonstrated substantial improvement after a five-day course [3,4,8-11]. Given the high relapse rate and inflammatory burden in POMS, early referral to a specialized MS center is essential to initiate disease-modifying therapy and coordinate neuro-ophthalmologic follow-up, rehabilitation, and psychosocial support [3,4,11].

## Conclusions

Persistent vertigo and gait instability in an adolescent, particularly when accompanied by central nystagmus, focal neurologic symptoms, or a history of prior unexplained neurologic episodes, should prompt consideration of POMS. Brain MRI demonstrating periventricular and callosal lesions with Dawson-finger morphology, along with spinal cord involvement and CSF evidence of intrathecal IgG synthesis, can rapidly establish the diagnosis and exclude key mimics. Timely treatment of acute relapses with high-dose corticosteroids and expedited referral to a tertiary MS center for long-term disease-modifying therapy are critical steps to optimize outcomes.
